# Supplementary figures and images for: Isolation of small extracellular vesicles from small volumes of blood plasma using size exclusion chromatography and density gradient ultracentrifugation
Source: eLife. 2026 May 22;13:RP92796. doi: 10.7554/eLife.92796 (PMC13197162; doi:10.7554/eLife.92796)

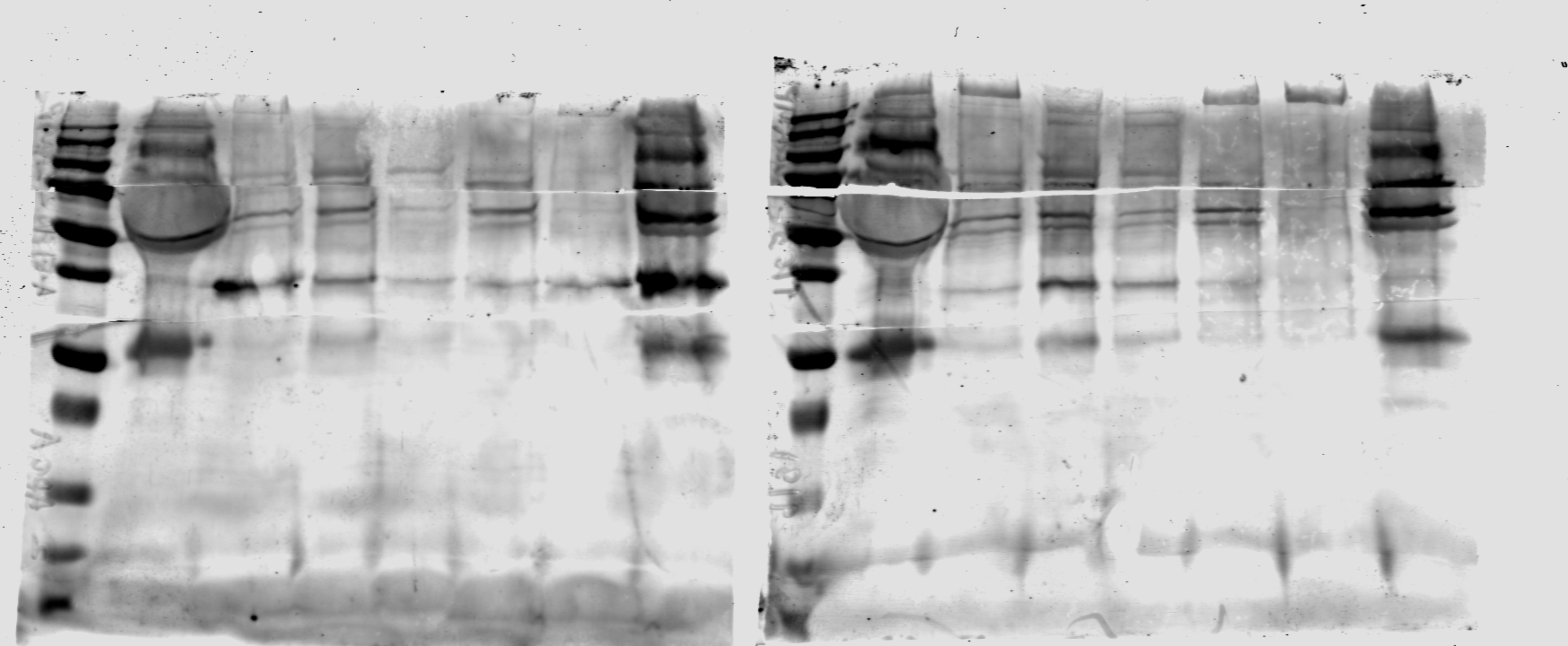

Supplement: Figure 6—source data 1. [file elife-92796-fig6-data1.zip › Figure 6-source data1/Figure6_CD81_raw.tif]

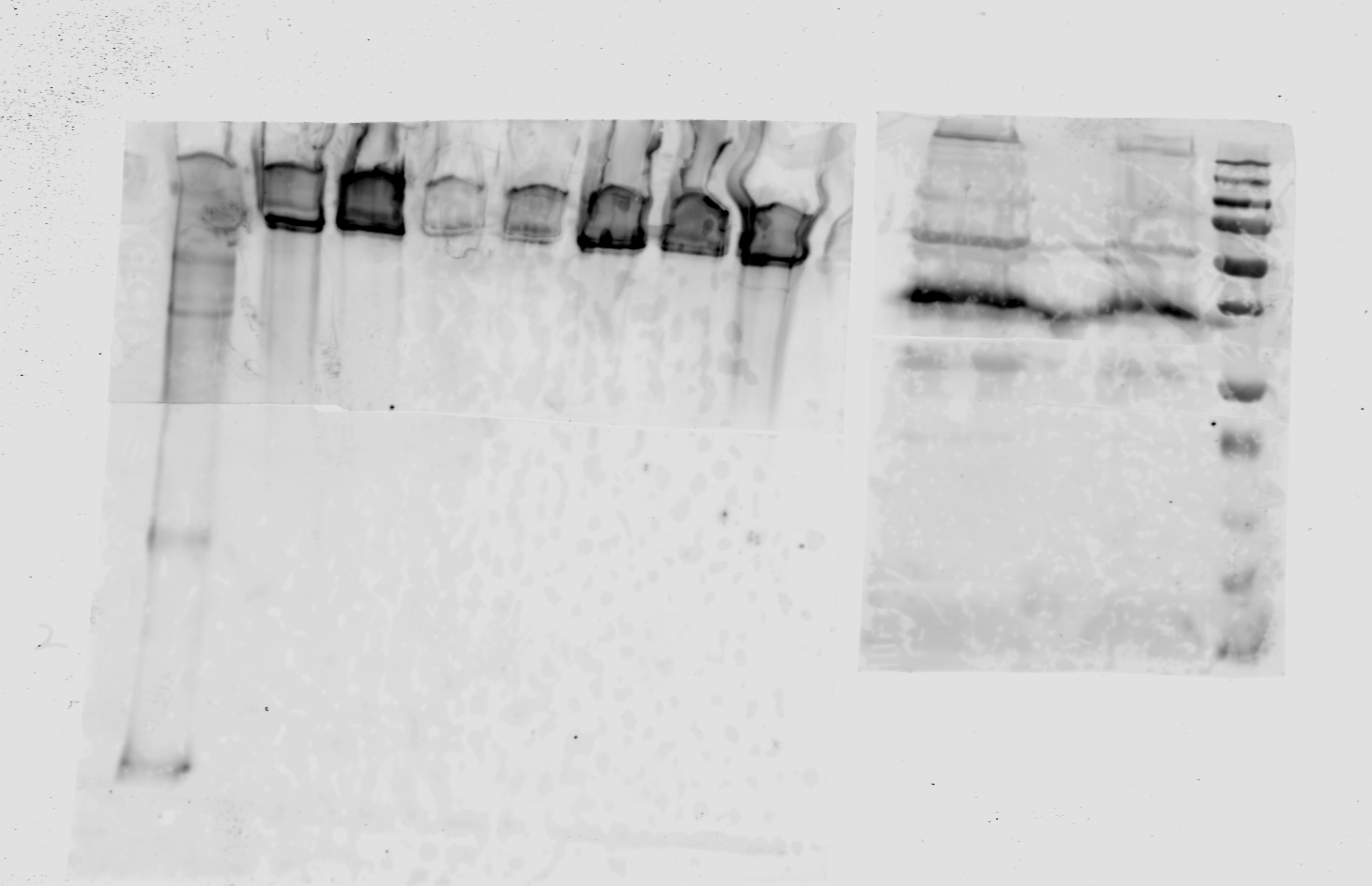

Supplement: Figure 6—source data 1. [file elife-92796-fig6-data1.zip › Figure 6-source data1/Figure6_ApoB_raw.tif]

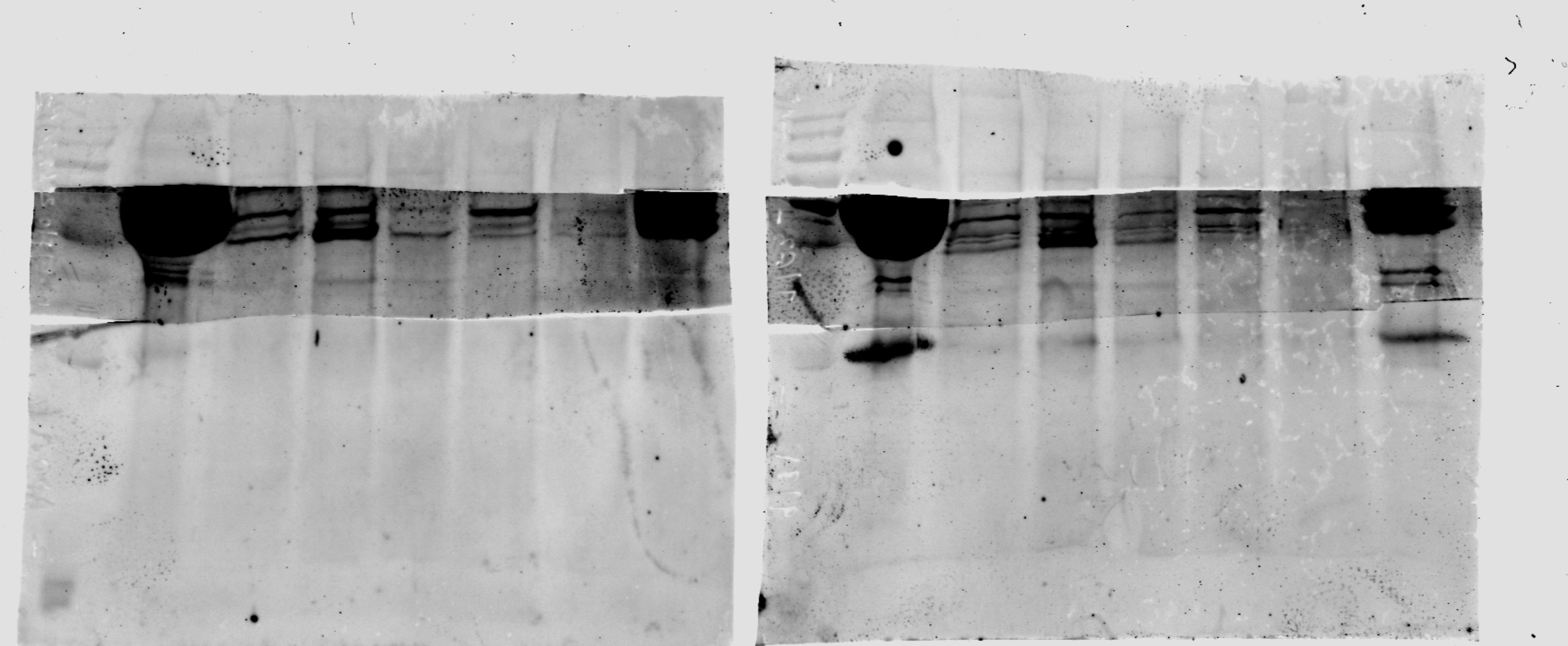

Supplement: Figure 6—source data 1. [file elife-92796-fig6-data1.zip › Figure 6-source data1/Figure6_CD9_raw.tif]

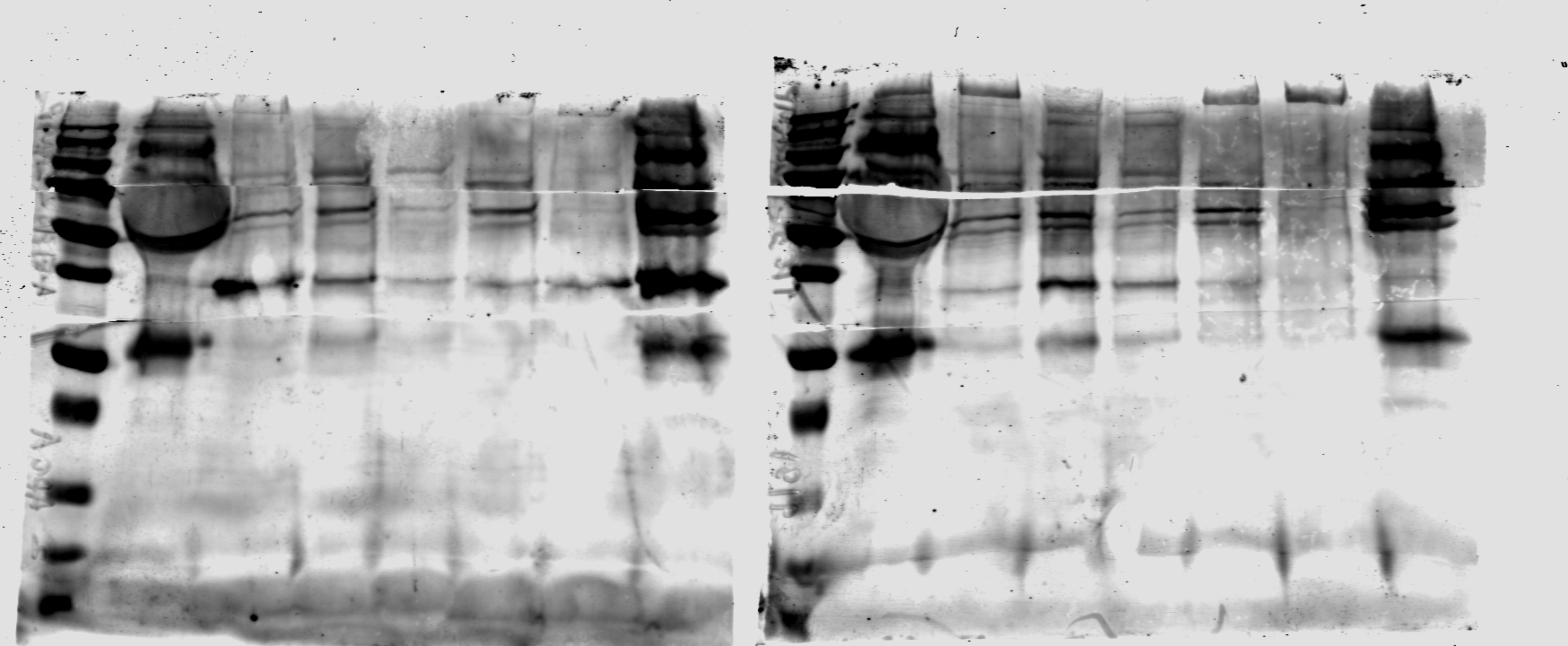

Supplement: Figure 6—source data 1. [file elife-92796-fig6-data1.zip › Figure 6-source data1/Figure6_CD63_raw.tif]

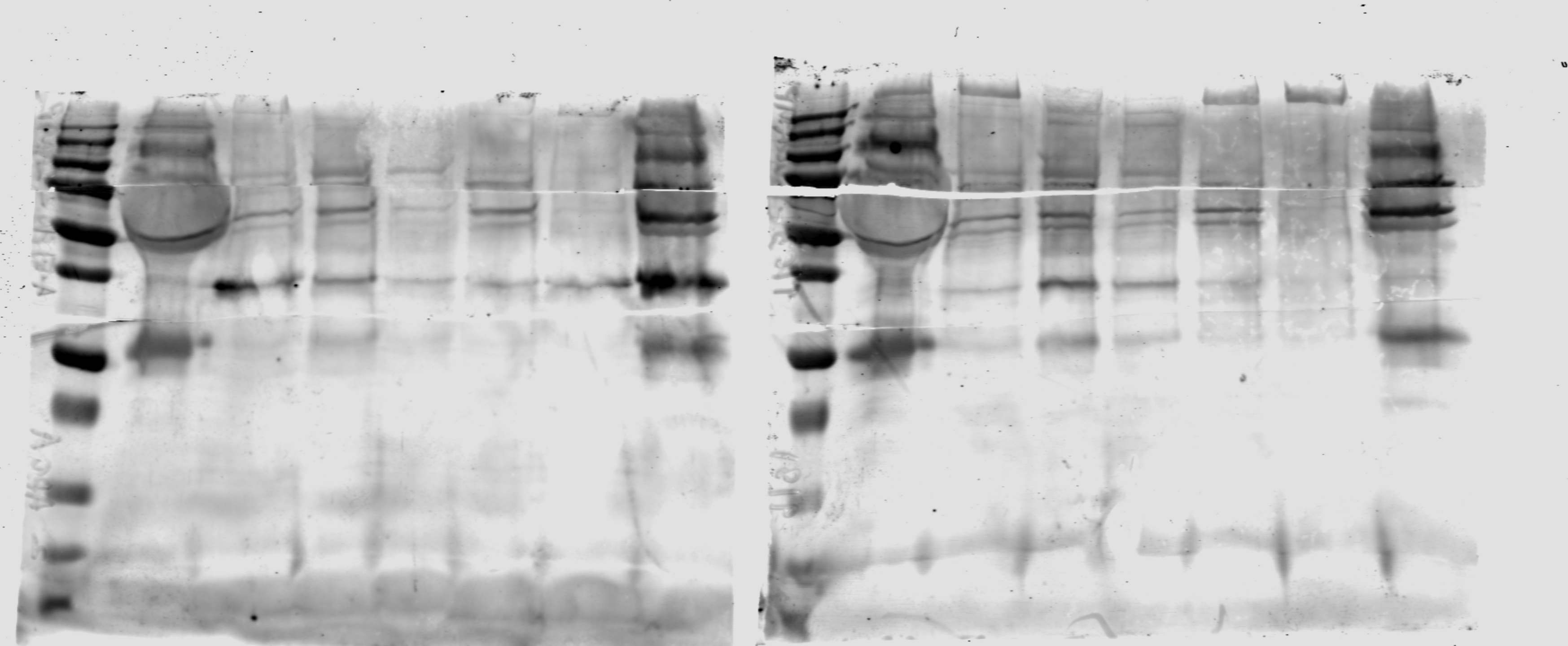

Supplement: Figure 6—source data 1. [file elife-92796-fig6-data1.zip › Figure 6-source data1/Figure6_ApoA_raw.tif]

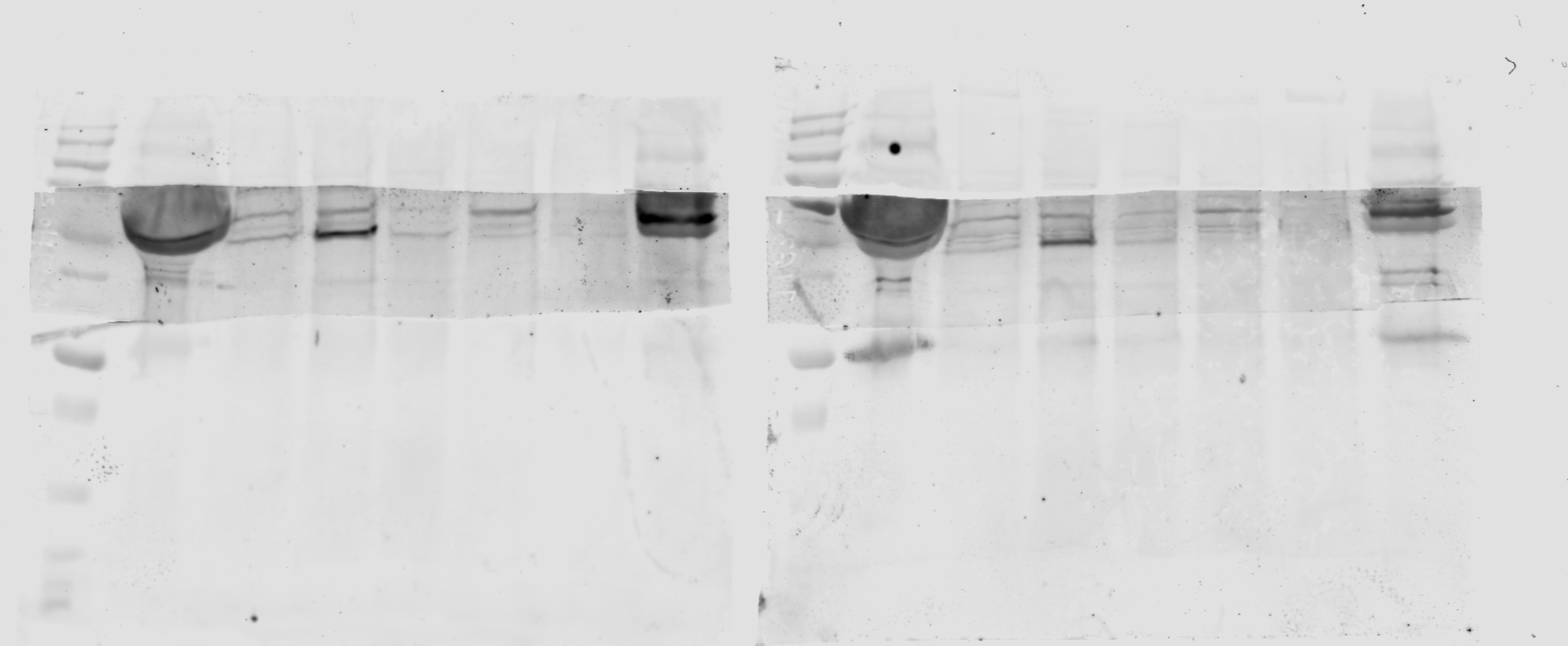

Supplement: Figure 6—source data 1. [file elife-92796-fig6-data1.zip › Figure 6-source data1/Figure6_TSG101_Flotillin_raw.tif]

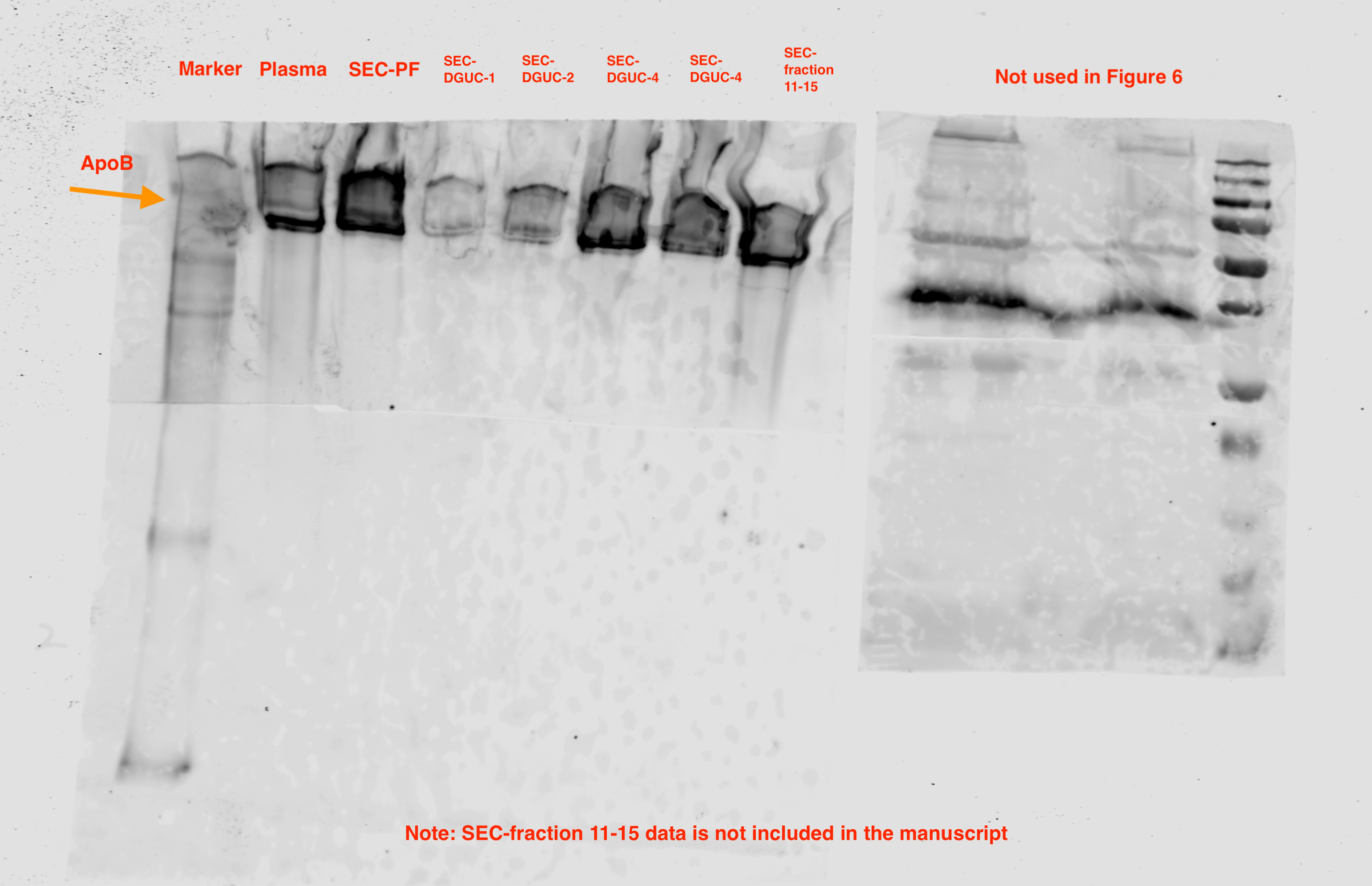

Supplement: Figure 6—source data 2. [file elife-92796-fig6-data2.zip › Figure 6-source data2/Figure6_ApoB_annotated.tif]

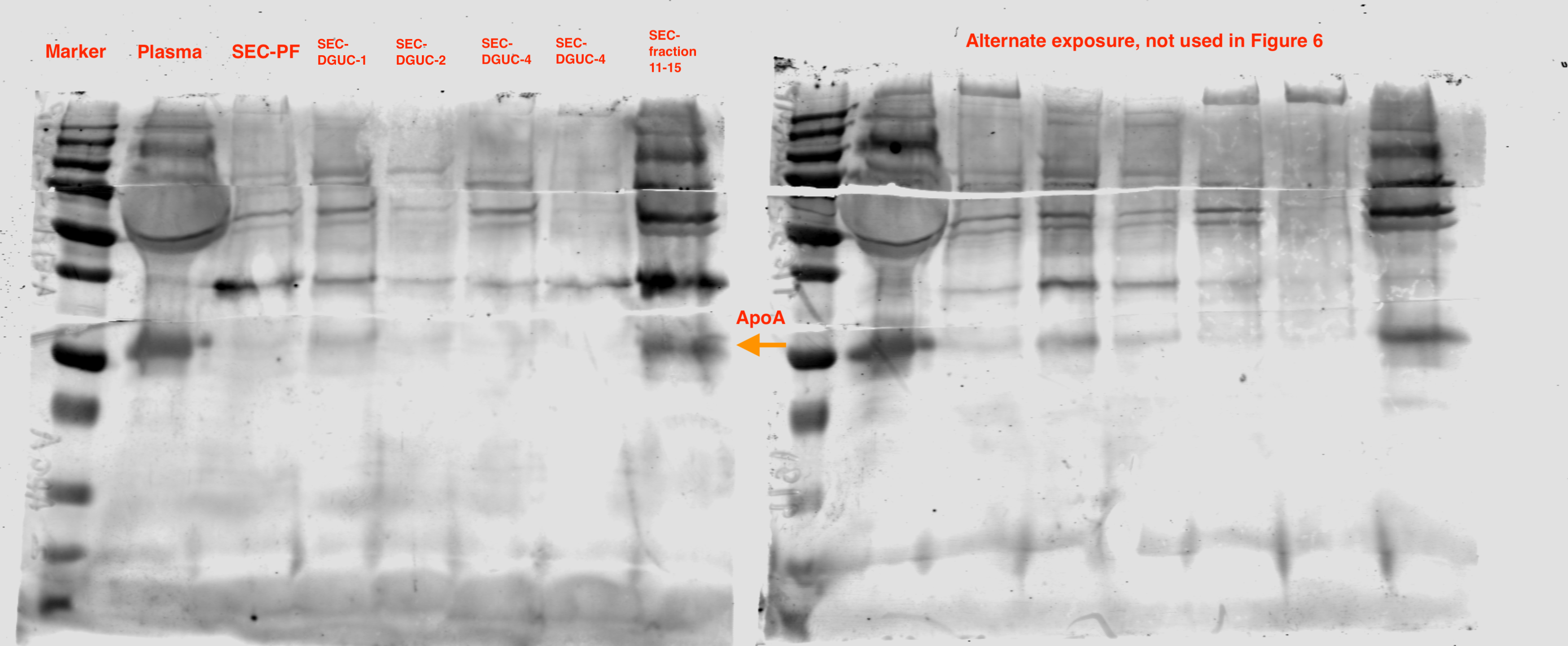

Supplement: Figure 6—source data 2. [file elife-92796-fig6-data2.zip › Figure 6-source data2/Figure6_ApoA_annotated.tif]

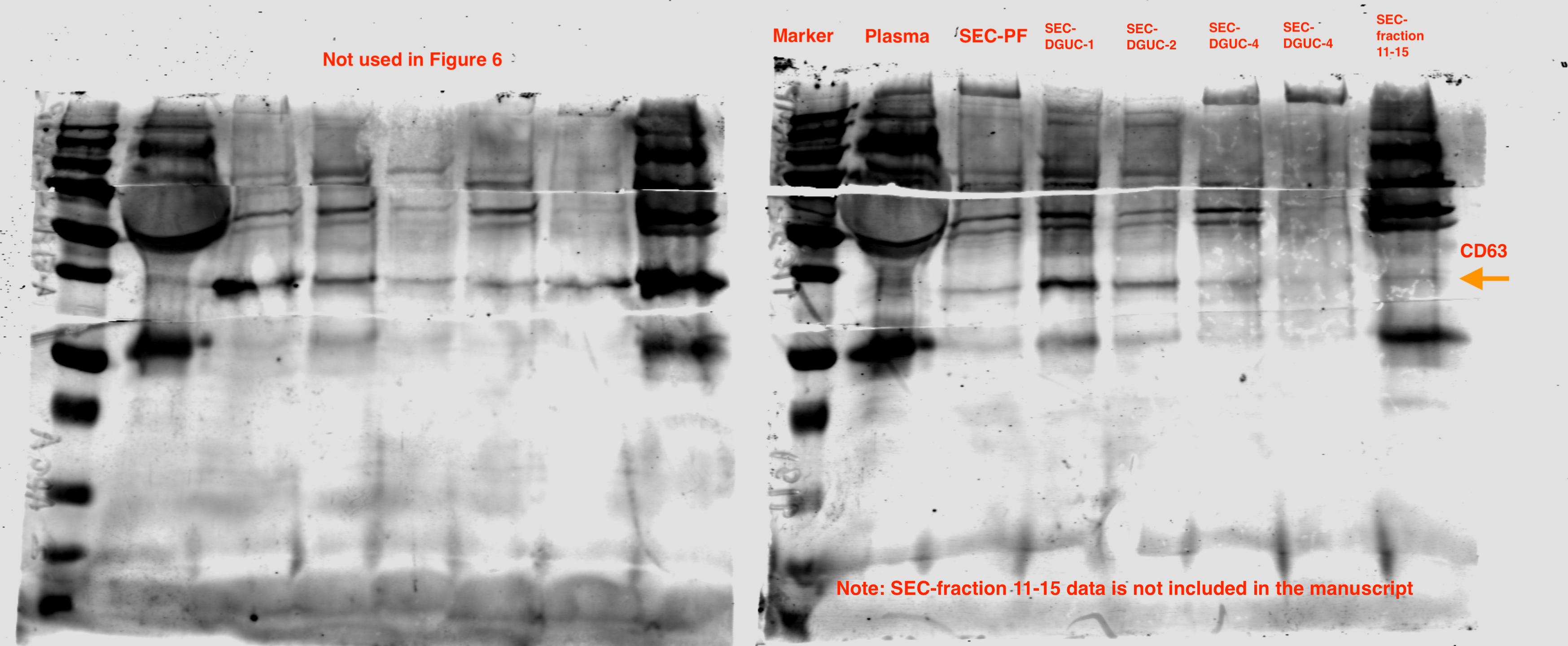

Supplement: Figure 6—source data 2. [file elife-92796-fig6-data2.zip › Figure 6-source data2/Figure6_CD63_annotated.tif.tif]

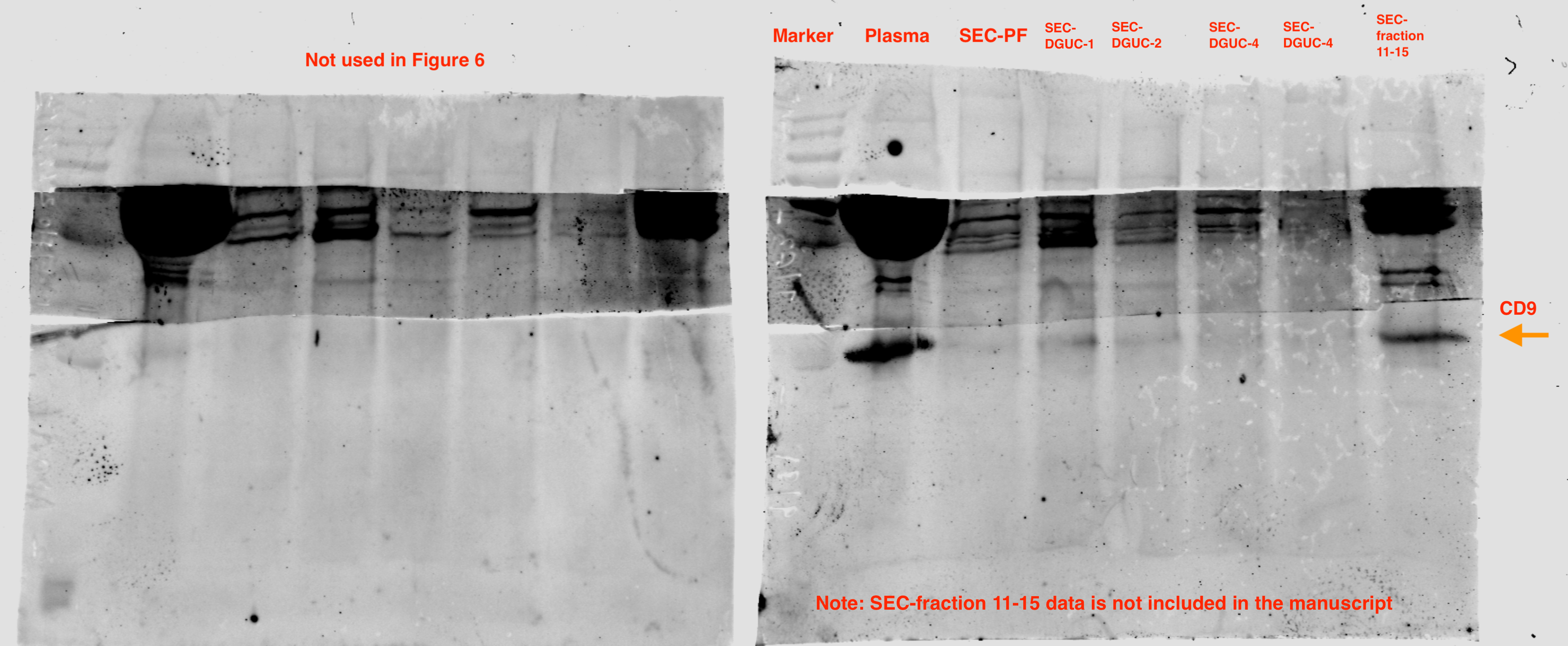

Supplement: Figure 6—source data 2. [file elife-92796-fig6-data2.zip › Figure 6-source data2/Figure6_CD9_annotated.tif]

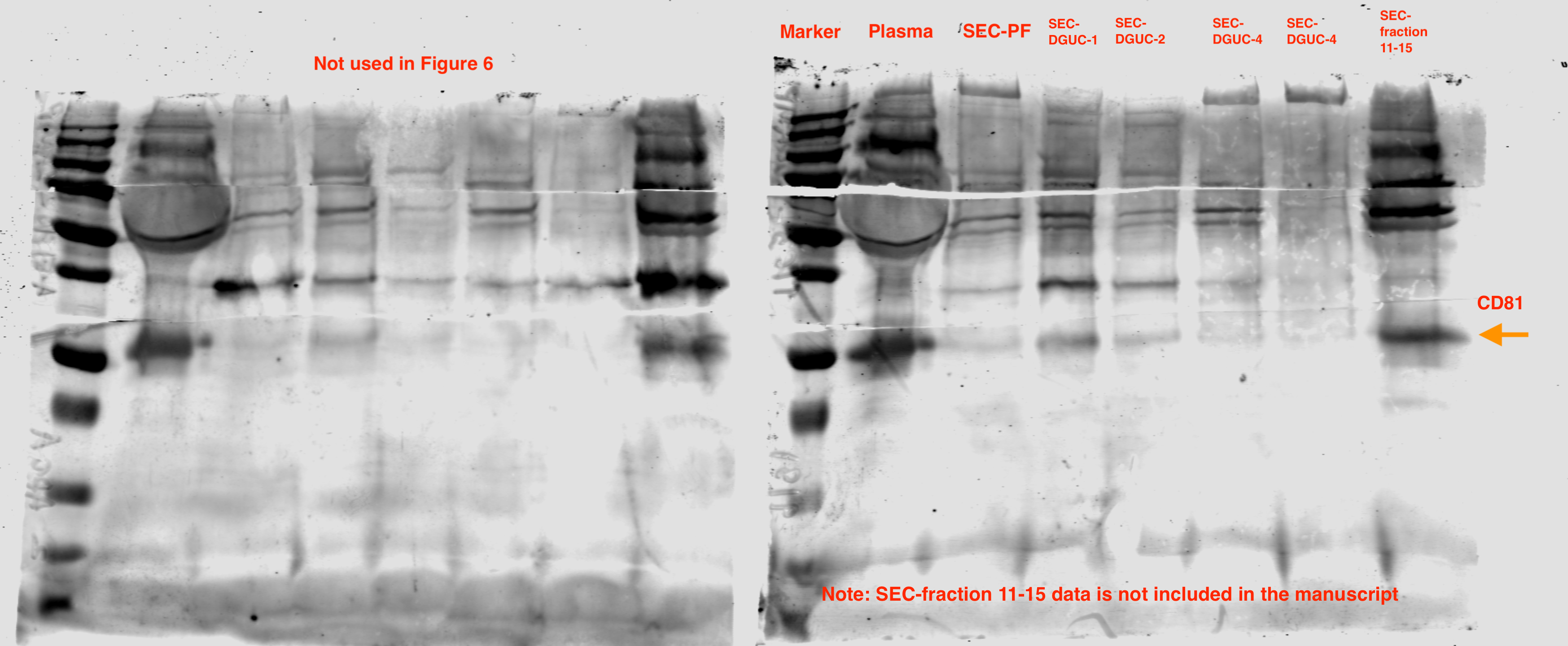

Supplement: Figure 6—source data 2. [file elife-92796-fig6-data2.zip › Figure 6-source data2/Figure6_CD81_annotated.tif]

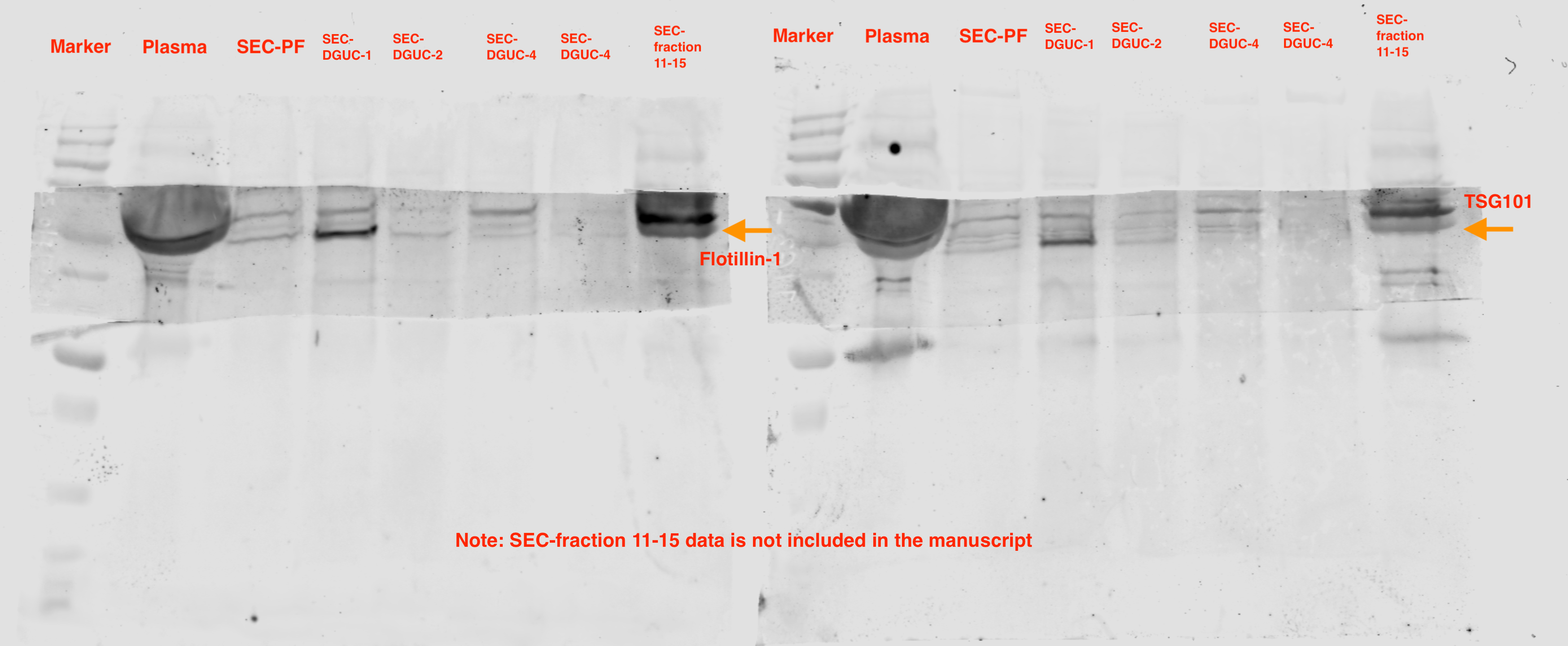

Supplement: Figure 6—source data 2. [file elife-92796-fig6-data2.zip › Figure 6-source data2/Figure6_TSG101_Flotillin-1_annotated.tif]

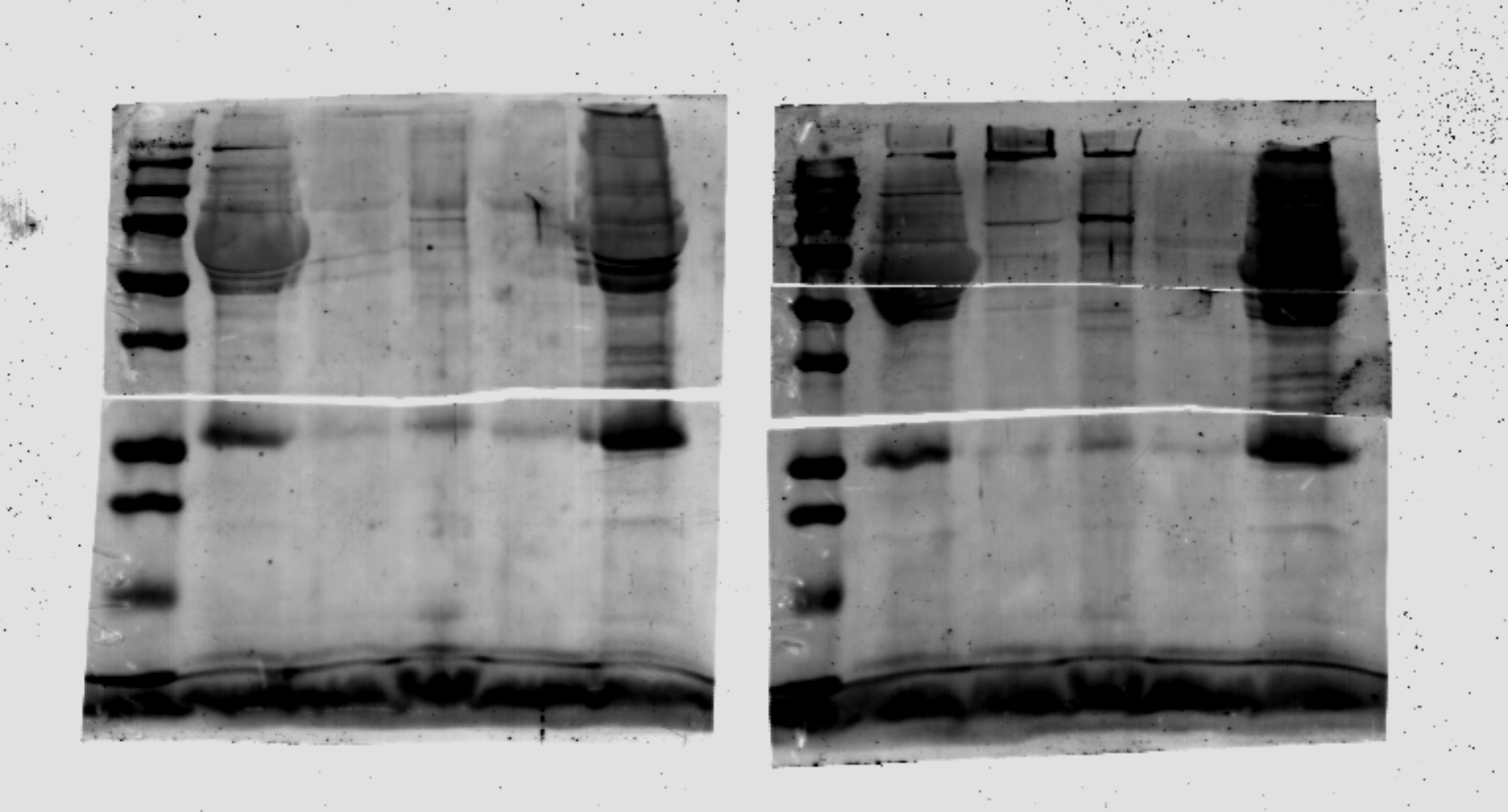

Supplement: Figure 7—source data 2. [file elife-92796-fig7-data2.zip › Figure 7-source data1/Figure7_CD9_raw.tif]

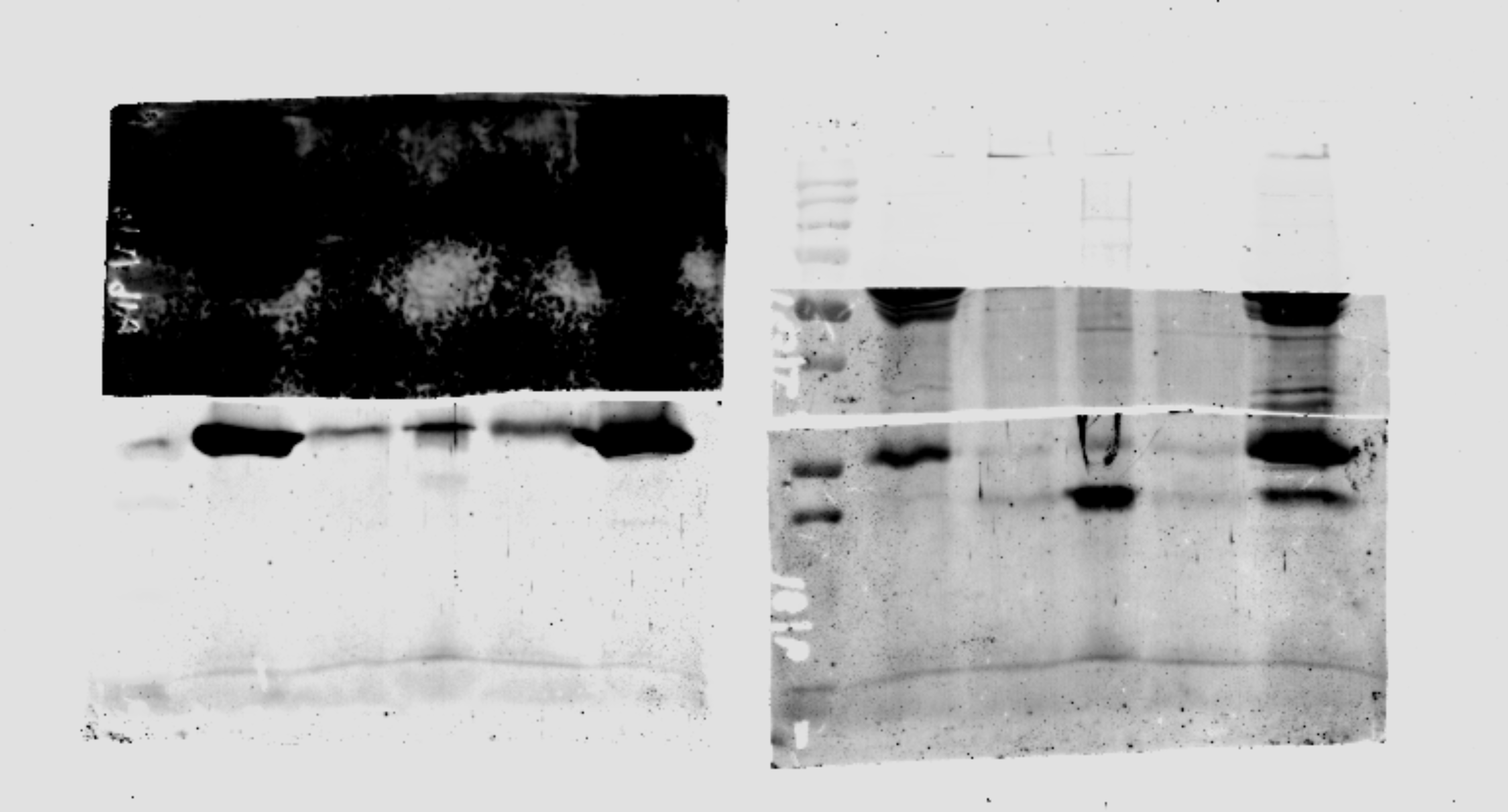

Supplement: Figure 7—source data 2. [file elife-92796-fig7-data2.zip › Figure 7-source data1/Figure7_CD81_raw.tif]

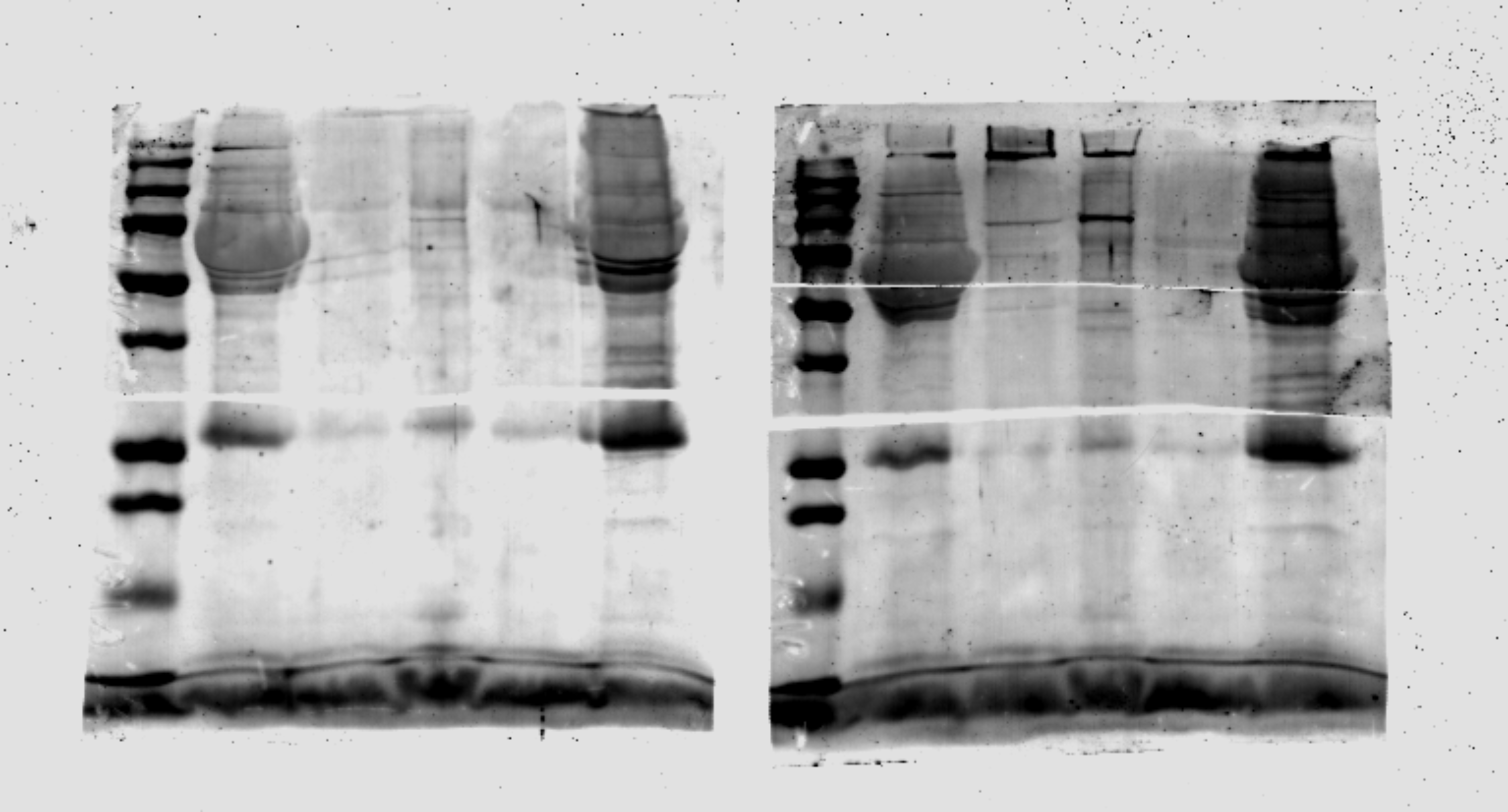

Supplement: Figure 7—source data 2. [file elife-92796-fig7-data2.zip › Figure 7-source data1/Figure7_TSG101_raw.tif]

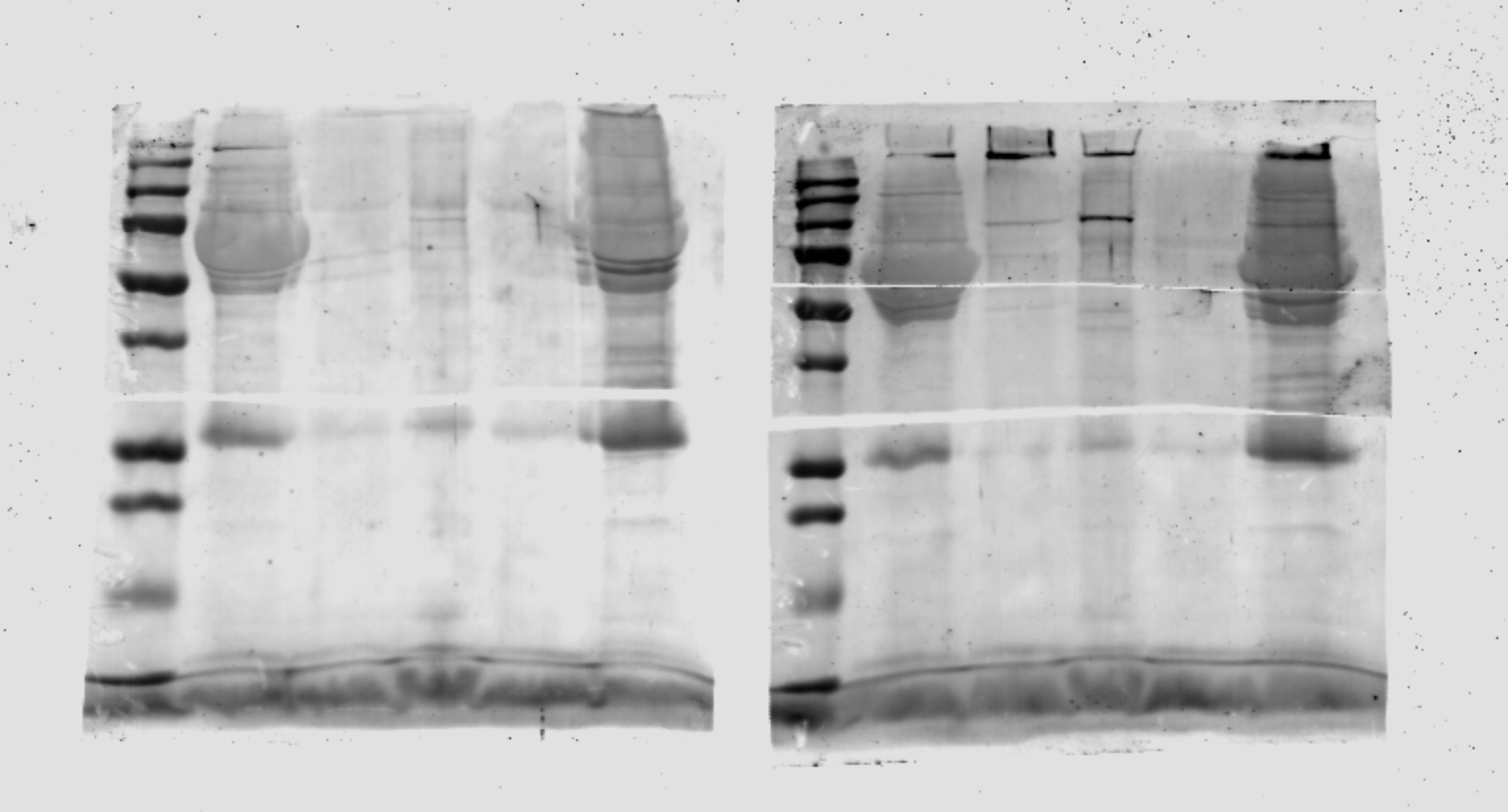

Supplement: Figure 7—source data 2. [file elife-92796-fig7-data2.zip › Figure 7-source data1/Figure7_Calnexin_raw.tif]

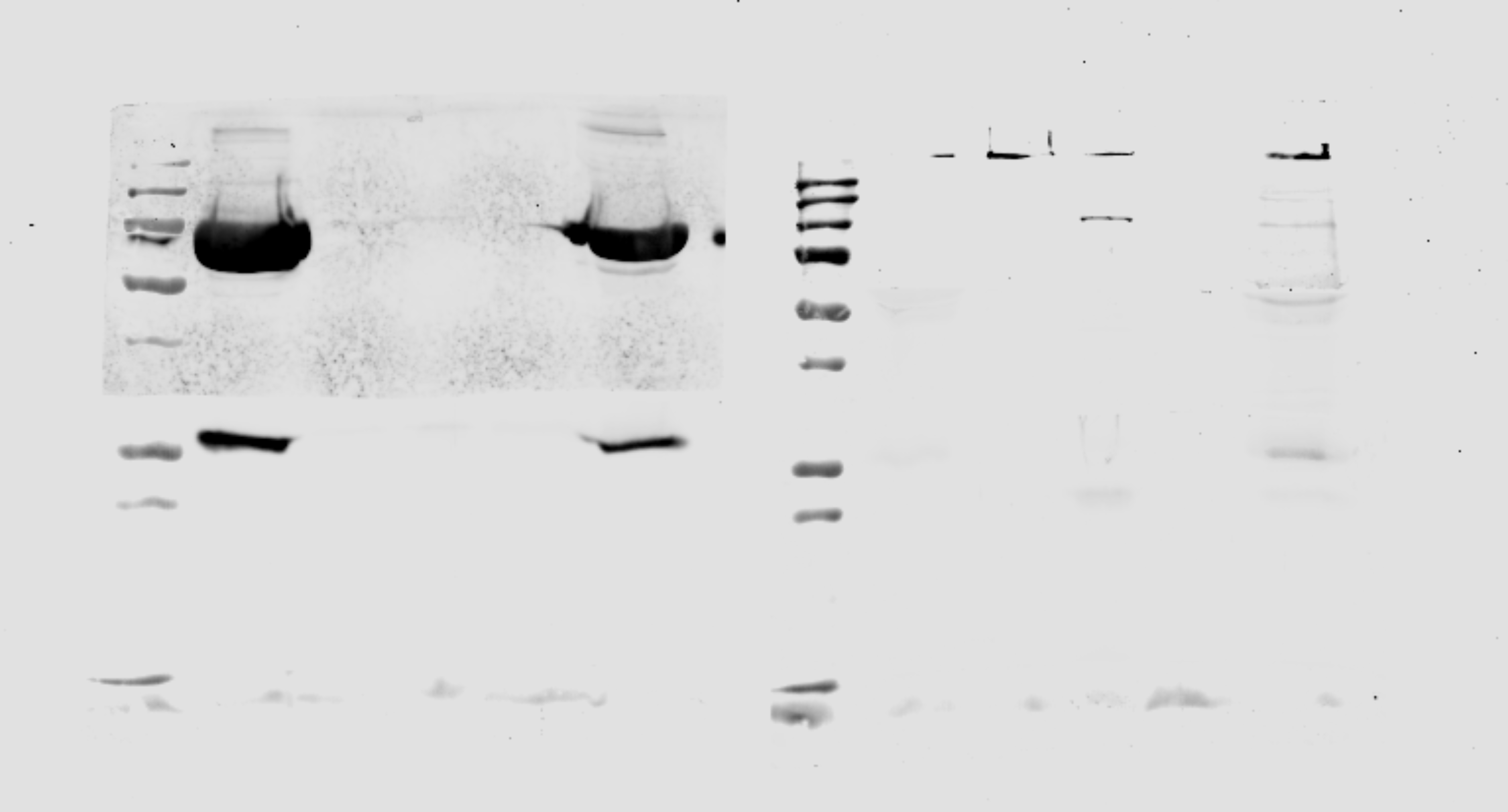

Supplement: Figure 7—source data 2. [file elife-92796-fig7-data2.zip › Figure 7-source data1/Figure7_Alubumin_raw.tif]

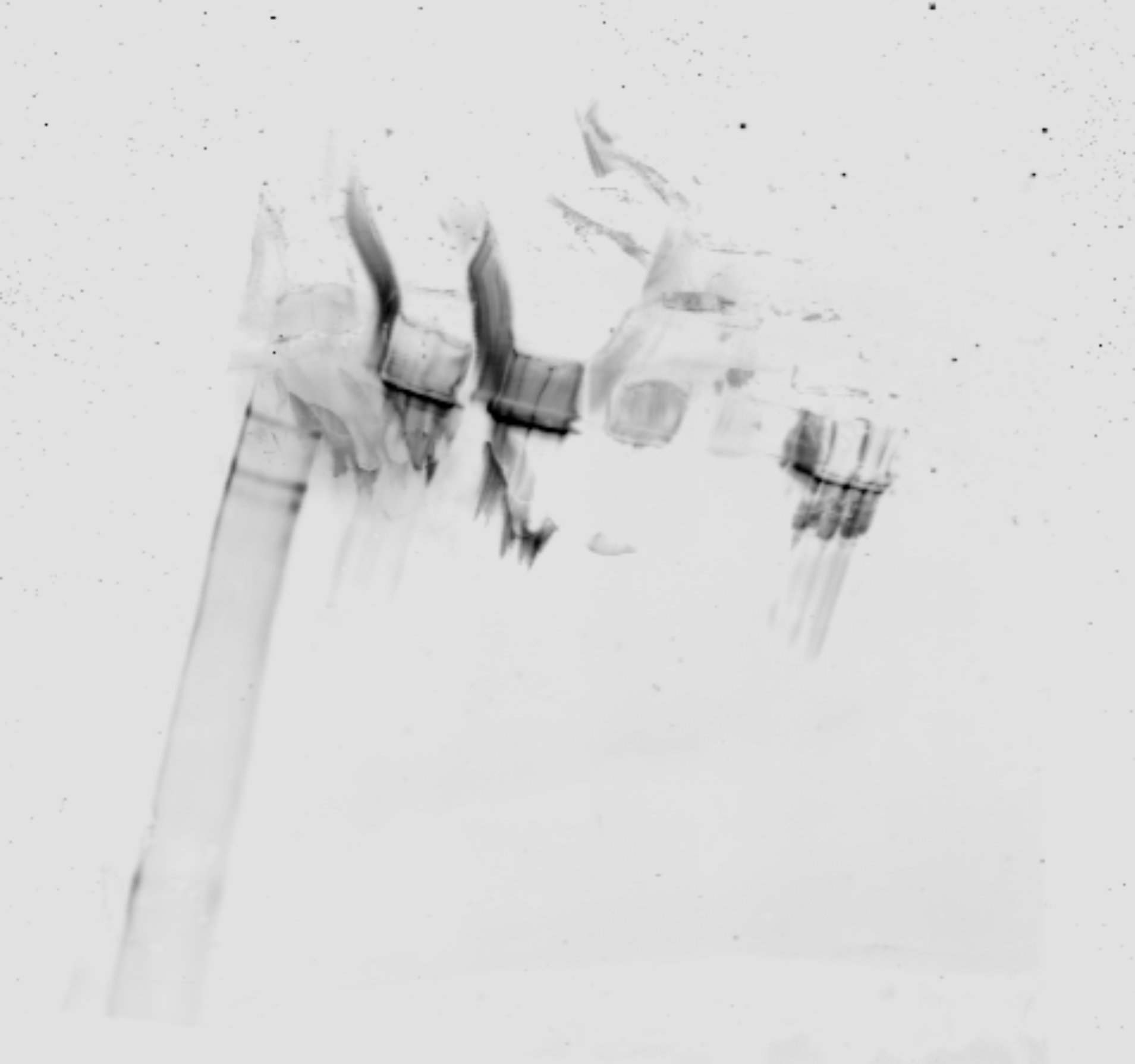

Supplement: Figure 7—source data 2. [file elife-92796-fig7-data2.zip › Figure 7-source data1/Figure7_ApoB_raw.tif]

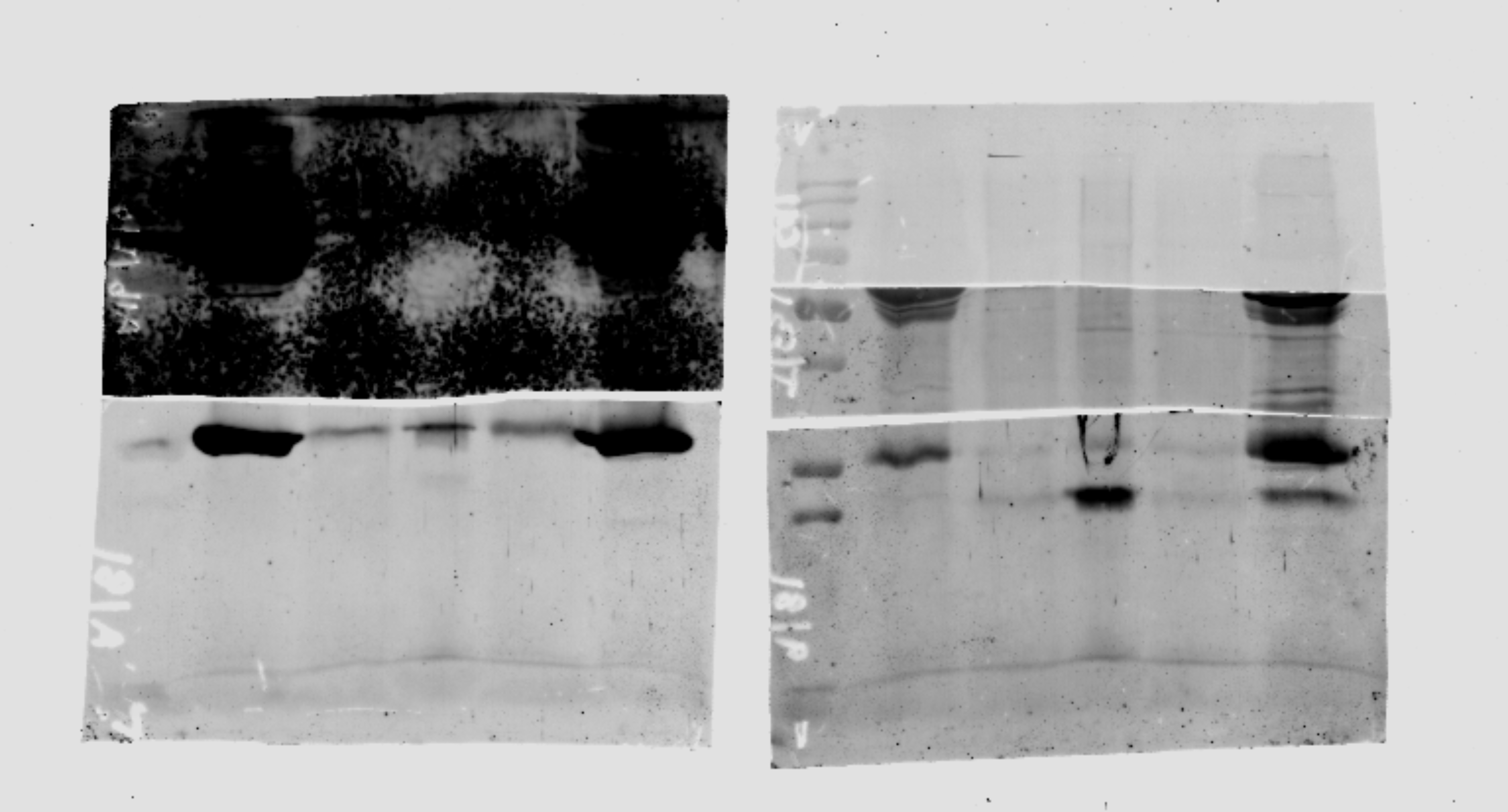

Supplement: Figure 7—source data 2. [file elife-92796-fig7-data2.zip › Figure 7-source data1/Figure7_CD63_raw.tif]

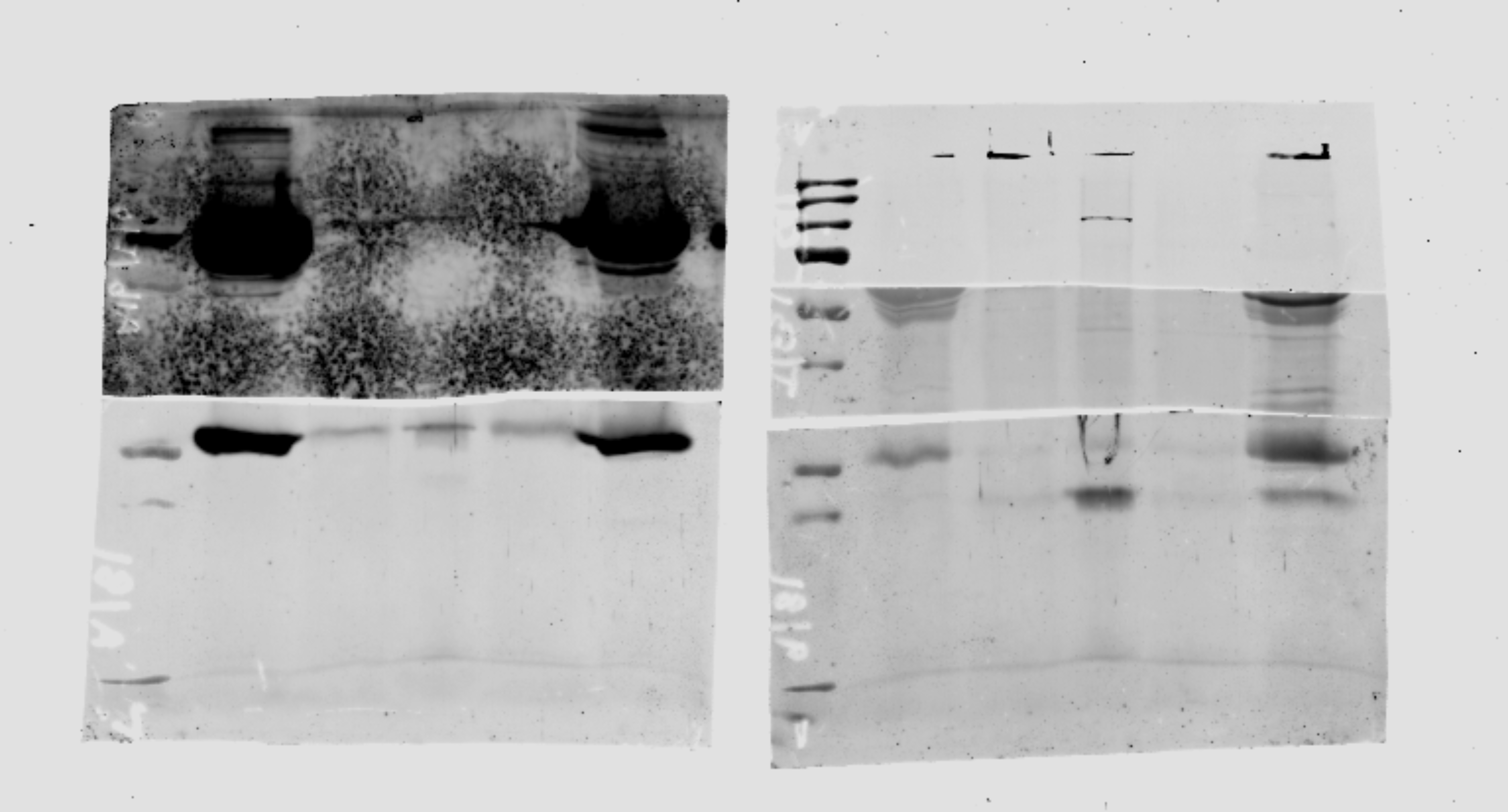

Supplement: Figure 7—source data 2. [file elife-92796-fig7-data2.zip › Figure 7-source data1/Figure7_ApoA_raw.tif]

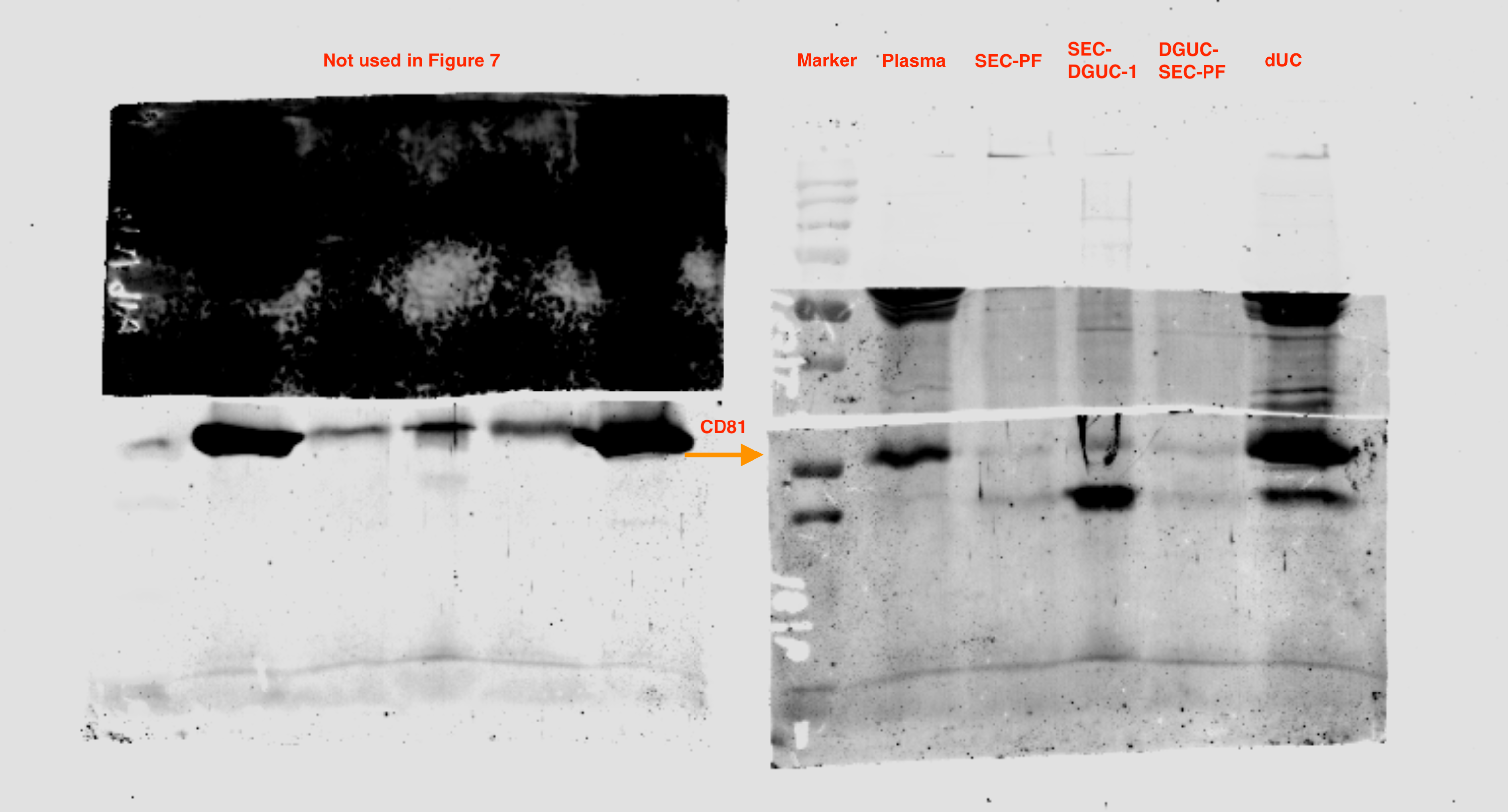

Supplement: Figure 7—source data 3. [file elife-92796-fig7-data3.zip › Figure 7-source data2/Figure7_CD81_annotated.tif]

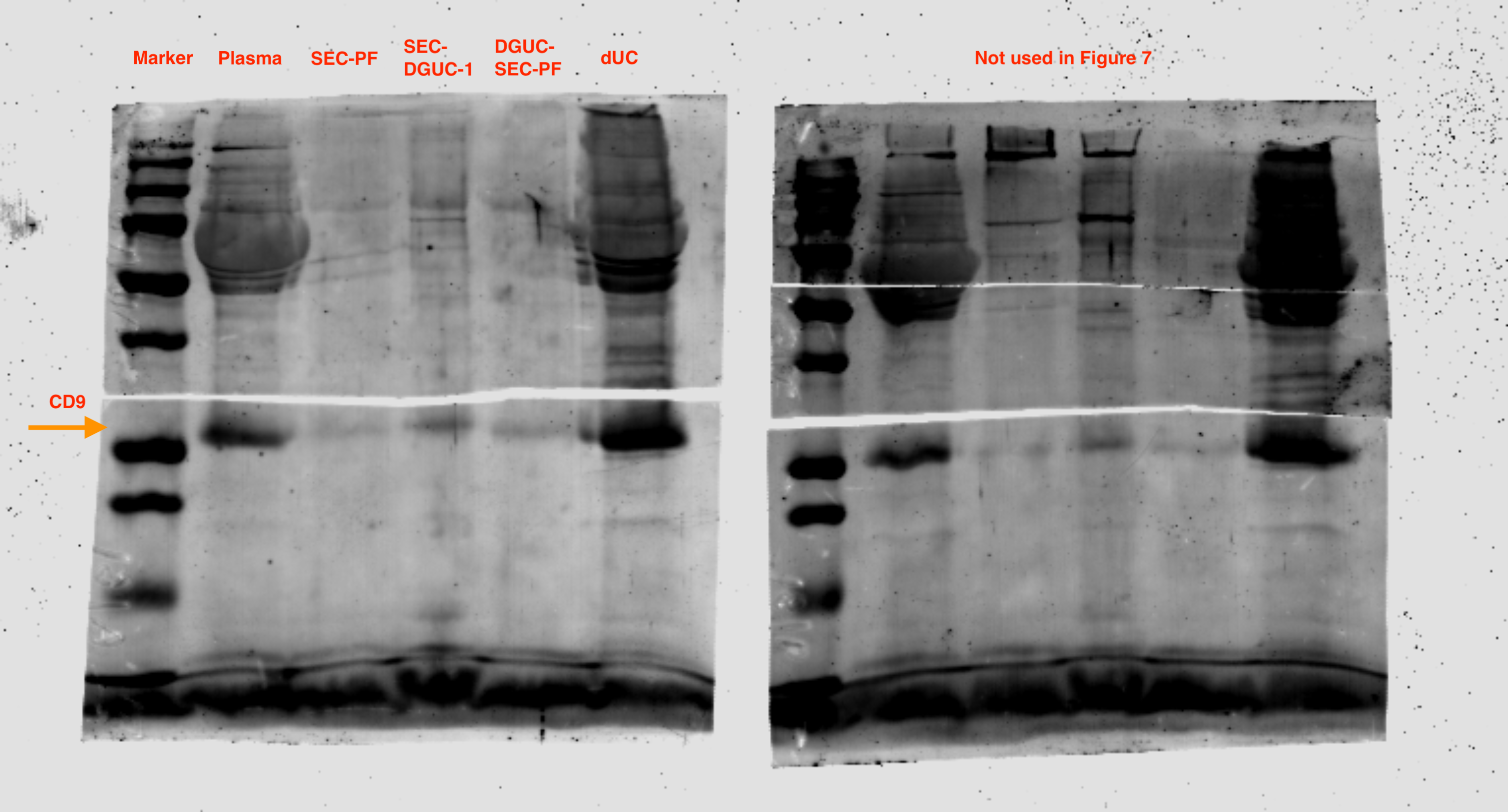

Supplement: Figure 7—source data 3. [file elife-92796-fig7-data3.zip › Figure 7-source data2/Figure7_CD9_annotated.tif]

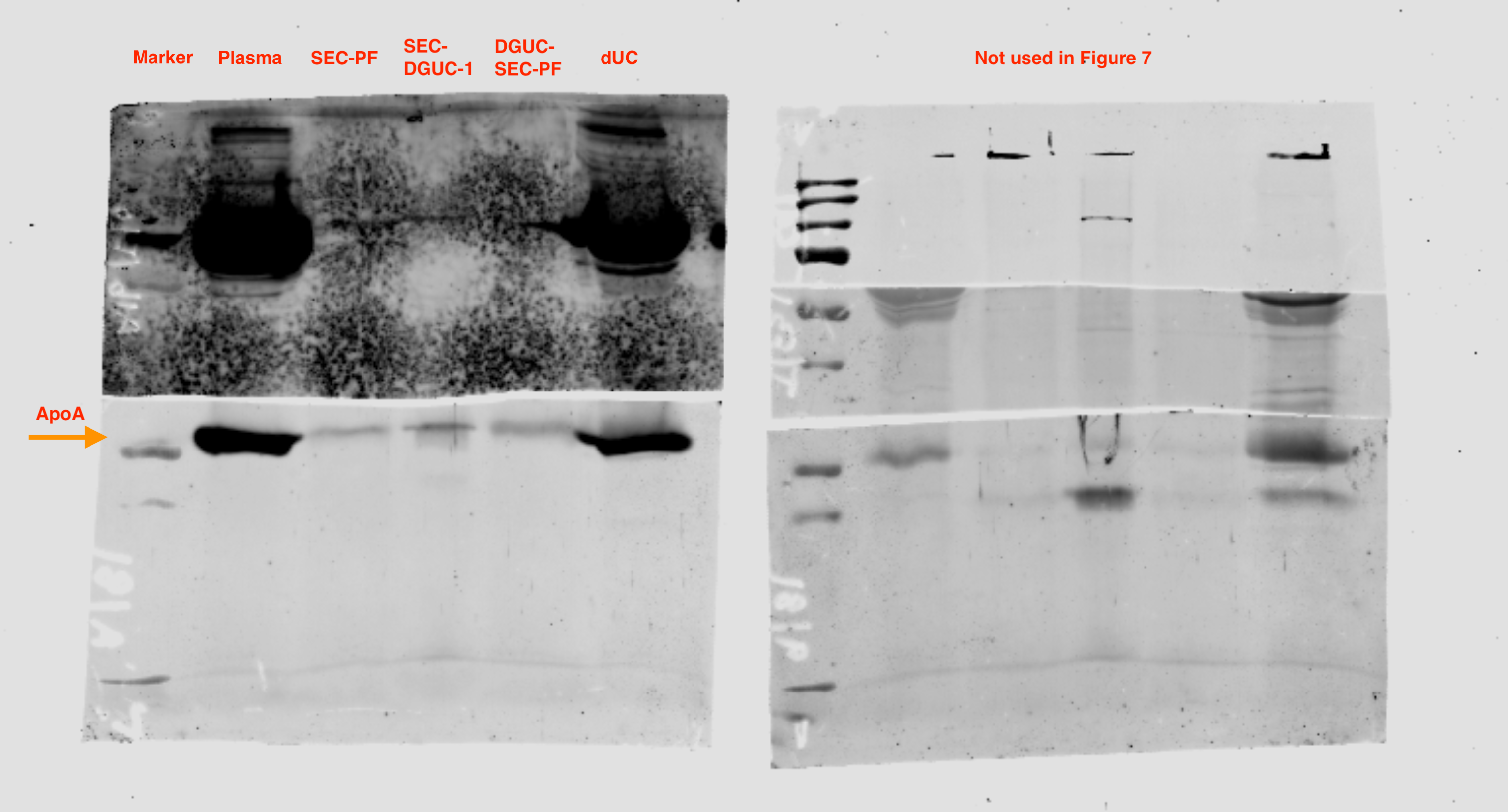

Supplement: Figure 7—source data 3. [file elife-92796-fig7-data3.zip › Figure 7-source data2/Figure7_ApoA_annotated.tif]

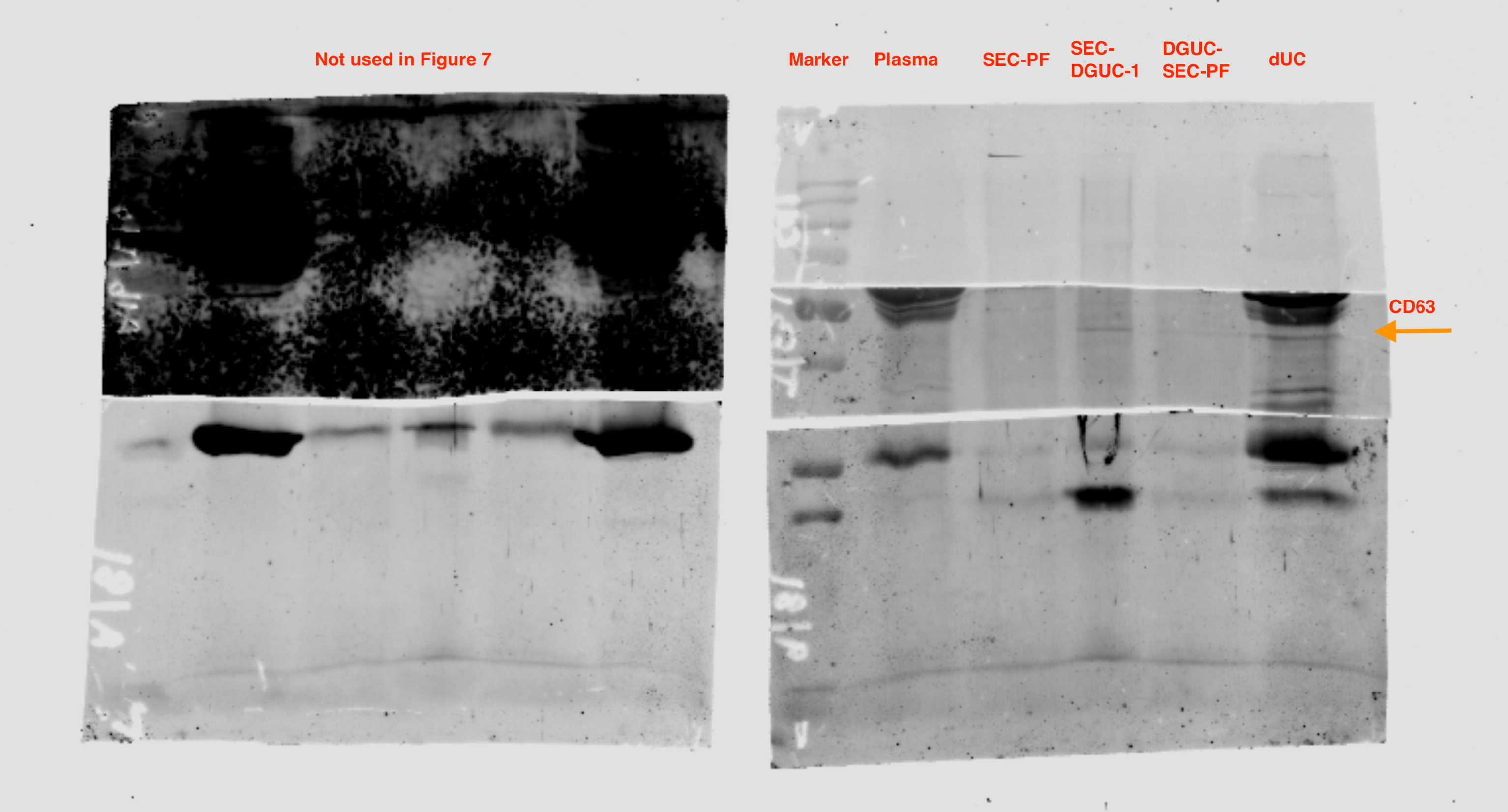

Supplement: Figure 7—source data 3. [file elife-92796-fig7-data3.zip › Figure 7-source data2/Figure7_CD63_annotated.tif]

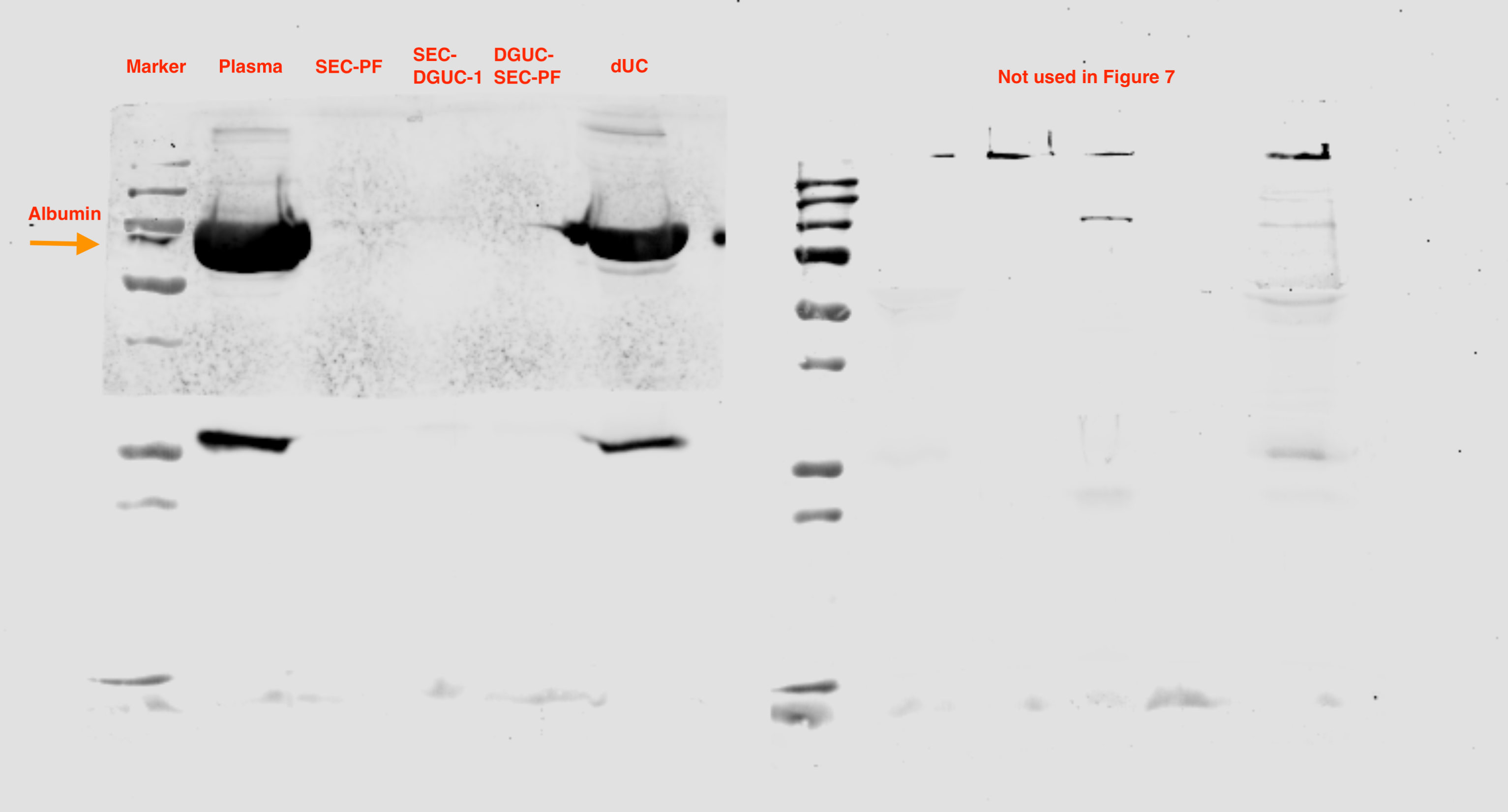

Supplement: Figure 7—source data 3. [file elife-92796-fig7-data3.zip › Figure 7-source data2/Figure7_Albumin_annotated.tif]

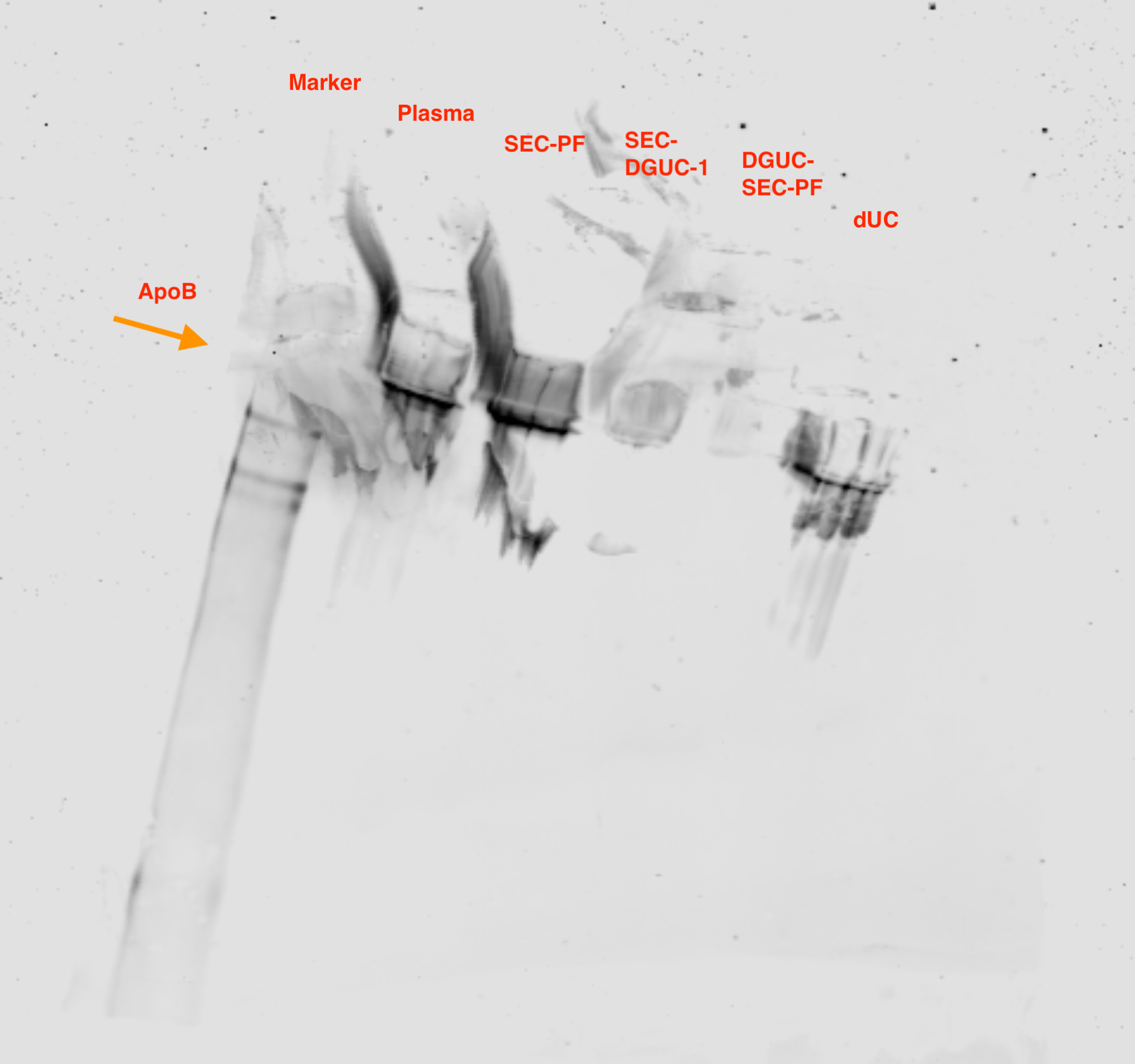

Supplement: Figure 7—source data 3. [file elife-92796-fig7-data3.zip › Figure 7-source data2/Figure7_ApoB_annotated.tif]

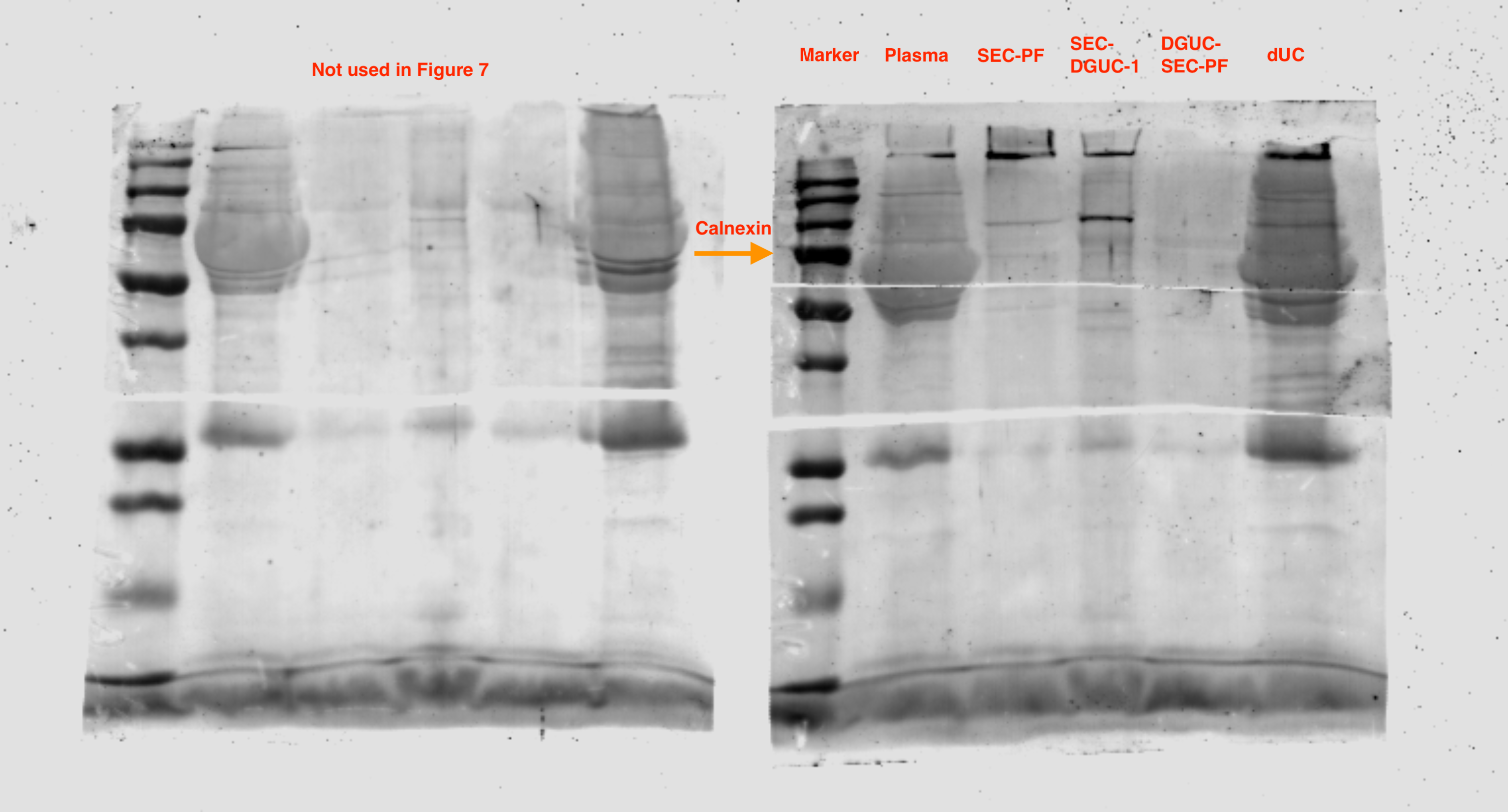

Supplement: Figure 7—source data 3. [file elife-92796-fig7-data3.zip › Figure 7-source data2/Figure7_Calnexin_annotated.tif]

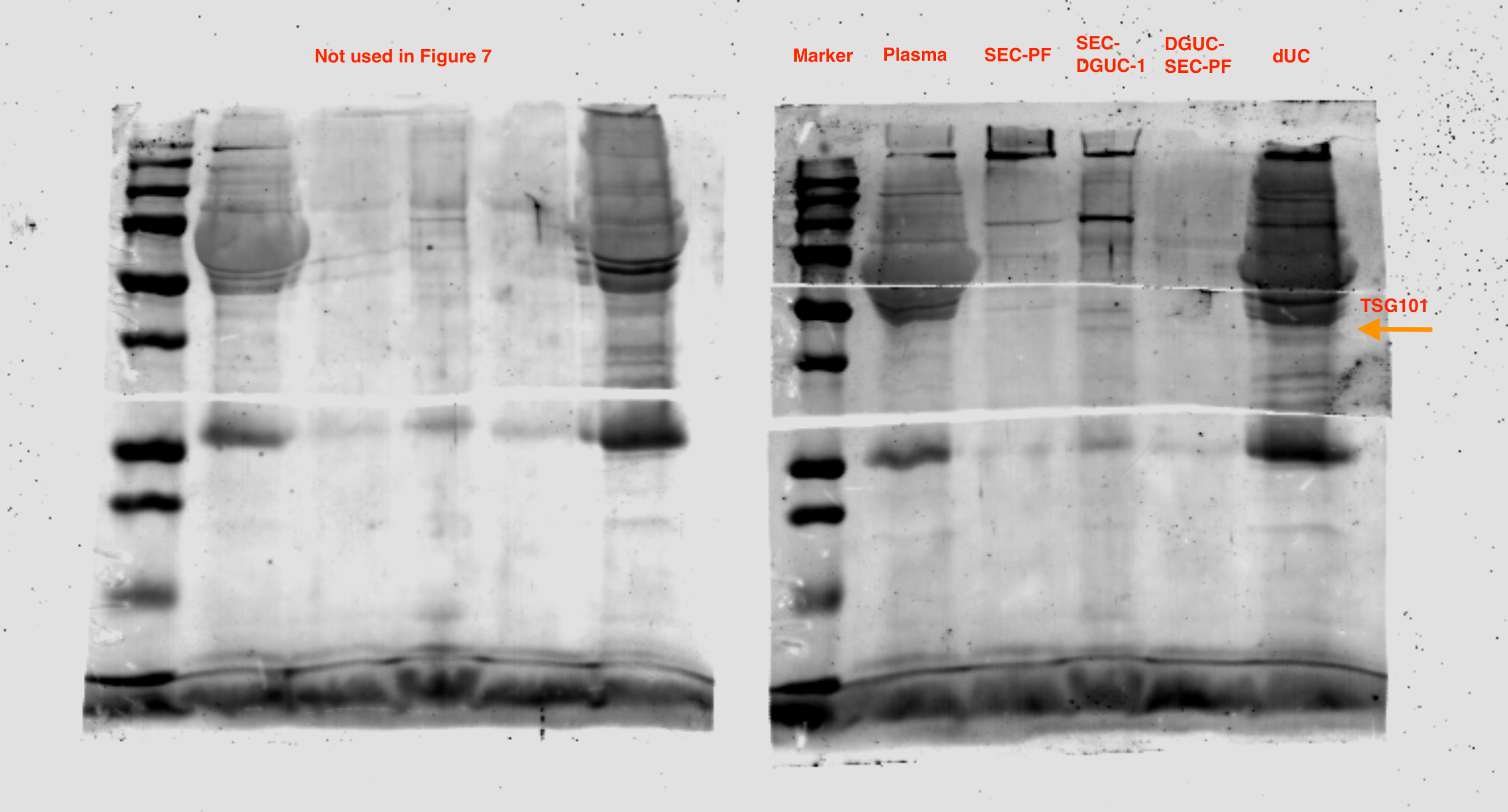

Supplement: Figure 7—source data 3. [file elife-92796-fig7-data3.zip › Figure 7-source data2/Figure7_TSG101_annotated.tif]
